# Bank1 and NF-kappaB as key regulators in anti-nucleolar antibody development

**DOI:** 10.1371/journal.pone.0199979

**Published:** 2018-07-17

**Authors:** Hammoudi Alkaissi, Said Havarinasab, Jesper Bo Nielsen, Peter Söderkvist, Per Hultman

**Affiliations:** 1 Molecular and Immunological Pathology, Department of Clinical Pathology and Clinical Genetics, Linköping University, Linköping, Sweden; 2 Department of Clinical and Experimental Medicine, Linköping University, Linköping, Sweden; 3 Institute of Public Health, Research Unit for General Practice, University of Southern Denmark, Odense C, Denmark; 4 Cell Biology, Department of Clinical and Experimental Medicine, Linköping University, Linköping, Sweden; University of Miami, UNITED STATES

## Abstract

Systemic autoimmune rheumatic disorders (SARD) represent important causes of morbidity and mortality in humans. The mechanisms triggering autoimmune responses are complex and involve a network of genetic factors. Mercury-induced autoimmunity (HgIA) in mice is an established model to study the mechanisms of the development of antinuclear antibodies (ANA), which is a hallmark in the diagnosis of SARD. A.SW mice with HgIA show a significantly higher titer of antinucleolar antibodies (ANoA) than the B10.S mice, although both share the same MHC class II (H-2). We applied a genome-wide association study (GWAS) to their Hg-exposed F2 offspring to investigate the non-MHC genes involved in the development of ANoA. Quantitative trait locus (QTL) analysis showed a peak logarithm of odds ratio (LOD) score of 3.05 on chromosome 3. Microsatellites were used for haplotyping, and fine mapping was conducted with next generation sequencing. The candidate genes *Bank1* (B-cell scaffold protein with ankyrin repeats 1) and *Nfkb1* (nuclear factor kappa B subunit 1) were identified by additional QTL analysis. Expression of the *Bank1* and *Nfkb1* genes and their downstream target genes involved in the intracellular pathway (*Tlr9*, *Il6*, *Tnf*) was investigated in mercury-exposed A.SW and B10.S mice by real-time PCR. *Bank1* showed significantly lower gene expression in the A.SW strain after Hg-exposure, whereas the B10.S strain showed no significant difference. *Nfkb1*, *Tlr9*, *Il6* and *Tnf* had significantly higher gene expression in the A.SW strain after Hg-exposure, while the B10.S strain showed no difference. This study supports the roles of *Bank1 (*produced mainly in B-cells) and *Nfkb1* (produced in most immune cells) as key regulators of ANoA development in HgIA.

## Introduction

Failure to recognize self with non-self-antigens results in a disorder of the innate and adaptive immune systems, leading to the development of autoantibodies, but the details of this process are still unclear [1]. Systemic autoimmune rheumatic disorder (SARD) is characterized by autoantibodies reactive with nuclear or subcellular organelles. It includes systemic lupus erythematosus (SLE), systemic sclerosis (SSc) and rheumatoid arthritis (RA). The prevalence and incidence of SARDs has increased during the last decade. Serum antinuclear antibodies (ANA) are used as serological markers in clinical practice and as laboratory tools in diagnostics of autoimmune diseases [2]. Systemic autoimmune disorders are triggered by genetic factors (such as MHC class II) [3], immunodeficiency [4], and environmental factors [5–8], making susceptible individuals more prone to developing the disease. Genome-wide association study (GWAS) is a tool for investigating genetic associations with autoimmune traits, and it is used to identify genetic risk factors for SARDs [9, 10].

Different animal models are used to study SARDs. Mercury-induced autoimmunity (HgIA) in mice is an established and relevant model, which includes the development of antinucleolar antibodies (ANoA), immune complex (IC) deposits, hypergammaglobulinemia and polyclonal B-cell activation, and is controlled by multiple genes [11–16]. One of them resides in the I-A region of the MHC class II locus (H-2). Mouse strains with haplotype H-2^*s*^ have the highest susceptibility for developing ANoA, while H-2^*q*^ and H-2^*f*^ mice have intermediate susceptibility, and H-2^*a*^, H-2^*b*^, and H-2^*d*^ mice are resistant to ANoA development [17].

However, knockout (KO) studies in mice have shown that non-H-2 genes also control the susceptibility to the development of systemic autoimmune disease [18–20]. HgIA in IL-6^-/-^, CD28^-/-^, and IFN^-/-^ H-2^*s*^ mice does not result in the development of ANoA [19, 20]. Additionally, strains sharing the same H-2^*s*^ show dissimilar severity of disease activity in HgIA. When comparing the two susceptible H-2^*s*^ strains, A.SW and B10.S, the A.SW strain shows a more severe autoimmune manifestation by developing a higher serum ANoA titer, higher IgG IC titer, and higher serum IgG1 and IgG2a titers compared to the B10.S strain [17, 21–23].

Crossing two strains with the same H-2^*s*^ (A.SW and B10.S) allowed us to investigate the non-H-2 genes, involved in the development of ANoA, by using GWAS. Mapping the quantitative trait loci (QTL) associated with an autoimmune trait was done with next generation sequencing (NGS), which allowed us to detect variants within the associated haplotype and identify the genes associated with the development of ANoA.

We identified a region on chromosome 3 in which the two genes, *Bank1* (B-cell scaffold protein with ankyrin repeats 1, produced mainly in B-cells [24]) and *Nfkb1* (nuclear factor kappa B subunit 1, produced in almost all cell types [25]) are potential key regulators of the development of ANoA. Discovering genetic risk factors associated with ANoA will provide the ability to make predictions of who is at an increased risk, investigate the underlying biological mechanisms of autoantibody production and support the knowledge-based development of new prevention and treatment strategies.

## Results and discussion

### Antinucleolar antibody formation is both H-2 and non-H-2 related

**Fig 1:**
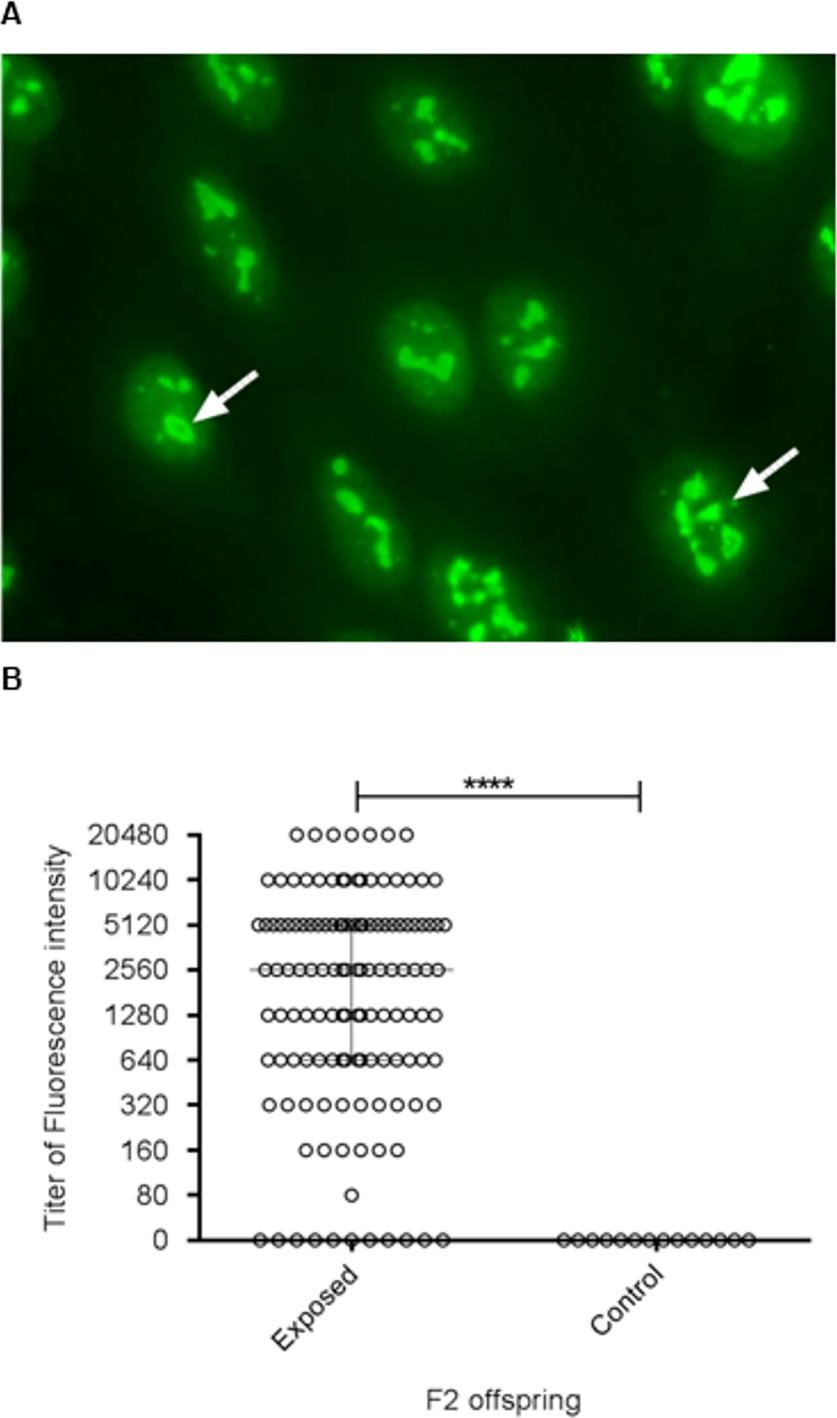
Serum antinucleolar antibodies (ANoA). Serum IgG ANoA in F2 mice control (n = 14) and F2 mice exposed to 4 mg HgCl_2_/L (n = 129) after 6-week exposure. A) ANoA assessed by indirect immunofluorescence using HEp2 cells. Arrows show strong clumpy staining of the nucleoli. B) Y-axis represents the ANoA titer (0–20,480). Graph is presented as the median ± interquartile range, ****p = < 0.0001 (Mann–Whitney test).

To achieve DNA recombination in F2 mice, we crossed two susceptible strains (A.SW and B10.S) sharing the same H-2 haplotype. The phenotypic trait and DNA recombination in F2 offspring were used as a tool for GWAS. The phenotypic trait ANoA corresponds to staining of the nucleoli with clumpy nucleolar pattern, with 2–6 brightly staining dots in the nucleoplasm (Fig 1A). The Hg-exposed F2 generation (n = 129) showed significantly higher ANoA titer (p = 0.0001) compared to control F2 mice (n = 14) (Fig 1B). We found a large inter-individual variation in exposed F2 mice indicating a genetic variation, consistent with non-H-2 genes regulating the development of ANoA [21].

### High ANoA titer is of an autosomal recessive inheritance

**Fig 2:**
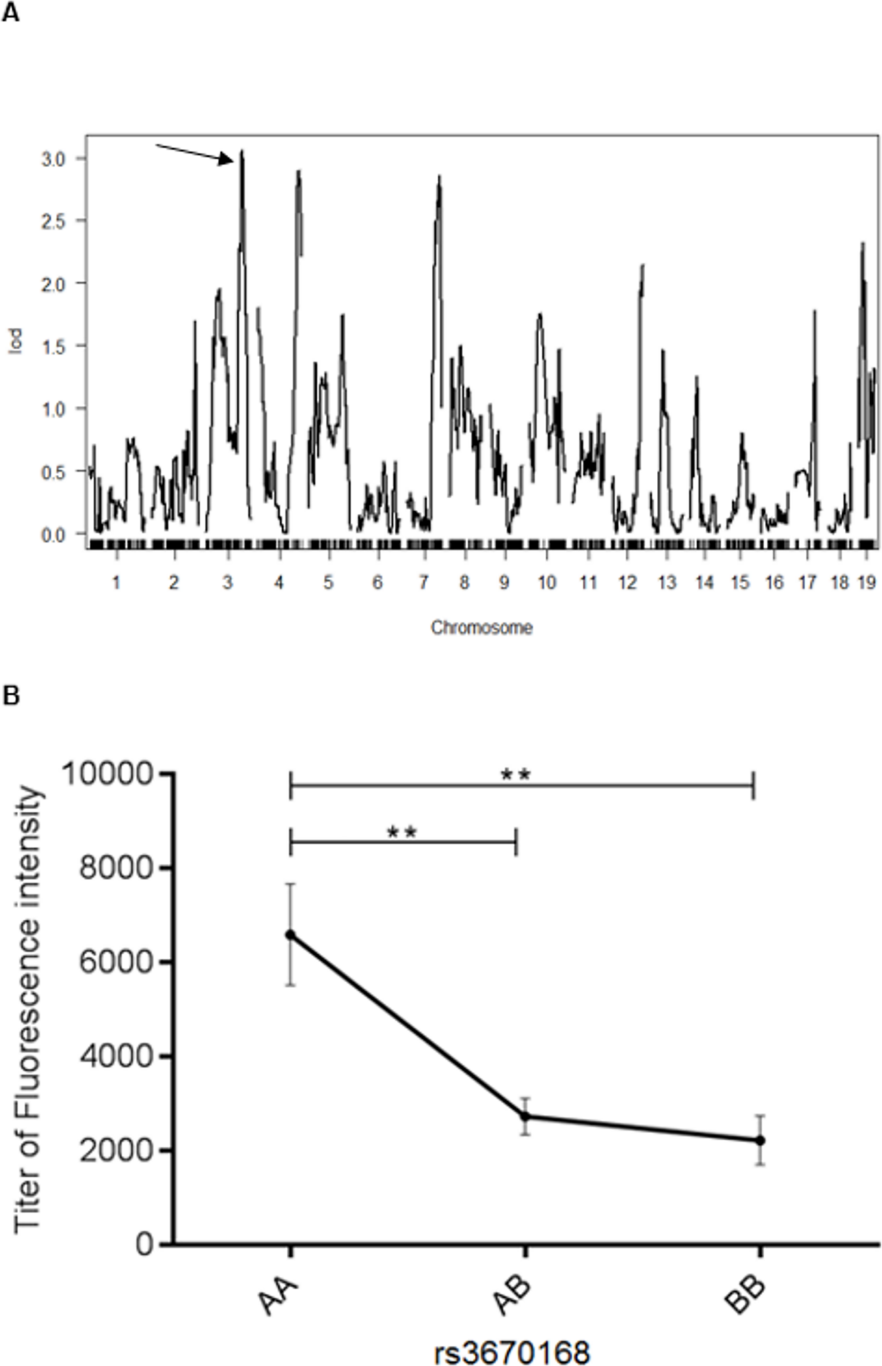
Genome-wide scan and effect plot. **A)** A genome-wide scan (n = 129) on autosomes was performed to identify quantitative trait loci associated with anti-nucleolar antibodies (ANoA) in mice exposed to mercury. Logarithm of odds (LOD) scores (y-axis) demonstrate curves over the murine autosomal chromosomes. X-axis demonstrates SNP markers on 19 autosomes. Lines represents association between genetic position and phenotype, serum antinucleolar antibodies. Arrow indicates the top peak on chromosome 3. **B)** Effect of different alleles in F2 offspring at peak marker rs3670168 on chromosome 3. Allele effects in the F2 offspring (X-axis), homozygous for A.SW (AA) or B10.S (BB) or heterozygous (AB) for ANoA titer (y-axis). The plot displays the mean ± SEM. **p < 0.01 (Mann-Whitney test).

Logarithm of the odds (LOD) scores exceeding 3.82, determined by permutation testing, represent approximate thresholds for significant QTLs based on the normal distribution with a p-value of 0.05 [26]. We found a QTL with the highest LOD score of 3.05 located at position 128292534 (rs3670168) on chromosome 3 (Fig 2A). The LOD score value showed a 99.9% linkage between the ANoA development and the position on chromosome 3. The inheritance outside H-2 suggests an autosomal recessive inheritance. Hanley and colleagues reported that crossing two strains, one resistant (H-2^*b*^) and one susceptible (H-2^*s*^) to developing ANoA, results in resistant F1 mice, suggesting a resistant dominant inheritance of the I-A region on H-2 [27]. We tested whether the ANoA phenotype was linked to the high or low autoantibody development by performing an effect plot. We found that F2 mice homozygous for the A.SW allele (AA) on the highest QTL (rs3670168) had a significantly higher ANoA titer than heterozygous mice (p < 0.01), or the mice homozygous for the B10.S allele (p < 0.01) (Fig 2B). F1 mice (A.SW x B10.S) had the same pattern of spreading between low and high serum ANoA titer (data not shown). Taken together, the inheritance requires the susceptible homozygote H-2^*s*^ loci, and the susceptibility for high ANoA titer is recessive.

### ANoA development is associated with *Bank1* and *Nfkb1*

**Fig 3:**
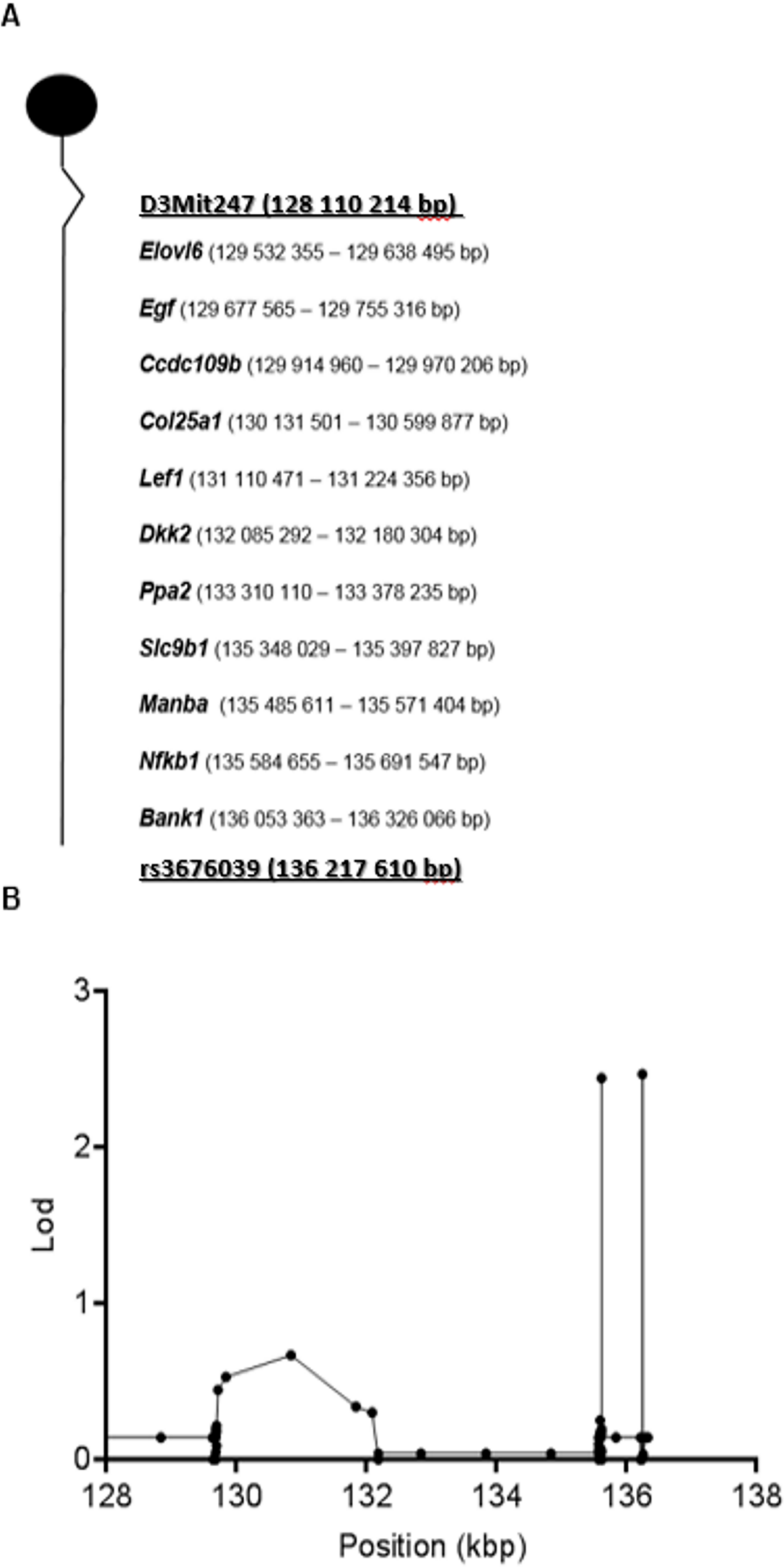
Fine mapping and QTL. **A**) Haplotypes associated with ANoA in the range 128 110 214–136 217 610 bp on chromosome 3, with 11 genes containing differences in SNPs between background strains A (for A.SW) and C57BL/6 (for B10.S). **B**) Fine mapping exons in 11 genes within the haplotype region performed with NGS to identify QTL associated with ANoA in 30 F2 mice homozygous for A.SW on rs3670168. LOD scores of 2.44 for *Nfkb1* and 2.46 and 2.47 for *Bank1*.

We narrowed down the QTL region with haplotype analysis by selecting Hg-exposed F2 mice homozygous for the A.SW allele on rs3670168. Selected F2 mice (n = 30) were genotyped with 9 additional microsatellite markers spaced between 54.48–61.32 cM. We found a haplotype block between D3Mit247 (128 110 214 bp) and rs3676039 (136 217 610 bp) (Fig 3A). We fine-mapped by sequencing the haplotype of the selected F2 mice, in eleven genes that contain SNPs between background strains A (for A.SW) and C57BL/6 (for B10.S). Unexposed A.SW and B10.S mice (n = 1 each) were also added as control mice for the analysis. Variants between B10.S and A.SW samples were extracted and compared with F2 variants, and we discovered 136 SNPs in total. To identify genes associated with the ANoA development, we performed additional QTL analysis by using the data on 136 SNPs and the phenotype data for 30 F2 mice. QTL association analysis of ANoA revealed 3 peaks, one in the *Nfkb1* gene (LOD 2.44) and two in the *Bank1* gene (LOD 2.46 and 2.47) (Fig 3B).

### *Bank1* mutations are missense variants and may alter protein structure

Bank1 is an adaptor/scaffolding protein expressed in most subpopulations of peripheral B-cells and has lower expression in plasmacytoid dendritic and myeloid cells but no expression in T-cells [24, 28]. Our next step was to investigate SNPs in *Bank1* between our two strains, A.SW and B10.S, by comparing them with the background strains (A and C57BL/6) using the Ensembl database [29]. We found 3 SNPs (rs30260564, rs50828248, and rs47442962) between background strains in which all SNPs are missense variants. rs30260564 resides in exon 2 and both rs50828248 and rs47442962 reside in exon 7 (Table 1). Amino acid changes may alter the structure, function, regulation and expression of a protein. We studied the secondary structure changes due to the missense variants. In the A.SW strain, CFFSP predicted [30] a β-sheet in the missense variants in exon 7 (rs50828248 and rs47442962, A375M), and this was not observed in the B10.S strain (S1 Fig). However, two other prediction algorithms did not show any changes in the secondary structure (data not shown). We believe that the structural difference between the two strains could affect the regulatory role that Bank1 has in ANoA development, but the prediction of secondary structures is limited using current mathematical models [31].

**Table 1:** Variants on *Bank1* gene.

ID (Ensembl)	Variant	Chr: bp	Exon	Transcript codon/amino acid A	Transcript codon/amino acid C57BL/6
rs30260564	Missense variant	3:136284103	2	TT**A** (Leu)	TT**C** (Phe)
rs50828248	Missense variant	3:136213910	7	**A**TG (Met)	**G**CG (Ala)
rs47442962	Missense variant	3:136213909	7	A**T**G (Met)	G**C**G (Ala)

*Nfkb1* is also associated with ANoA development, and we found 5 SNPs (rs13477428, rs30771025, rs13472038, rs31054249, and rs13472037) between background strains (A and C57BL/6), all with synonymous variants. There are different outcomes of silent mutations that, depending on their positions, may affect mRNA splicing, transcription and translation [32]. The SNPs in *Nfkb1* may have an impact on NF-kappaB gene/protein expression and function that may cause the ANoA titer differences.

### Bank1 SNP rs30260564 is conserved in the more susceptible strain

We investigated the conservation of the SNPs in Bank1 at the nucleotide and amino acid levels (see S2 Fig and S3 Fig). Two of the three SNPs in Bank1 (rs30260564 and rs50828248) are in a conserved region in the A strain but not in the C57BL/6 strain. rs30260564 corresponds to the codon TTA (leucine) in the A strain and to the codon TTC (phenylalanine) in the B10.S strain. All mammalian species code for the amino acid leucine, as observed in the A strain. The amino acid in the C57BL/6 strain is not conserved. The two SNPs rs50828248 and rs47442962 code for the same codon, ATG (methionine), in the A strain and GCG (alanine) in the C57BL/6 strain. Neither of these two mouse strains have their amino acids conserved. rs30260564 may be critical for vital organism function, and the substitution of phenylalanine for leucine (Phe75Leu) in exon 2 may be responsible for the high ANoA titer.

### Intracellular pathway in B-cells favors high ANoA titer

**Fig 4:**
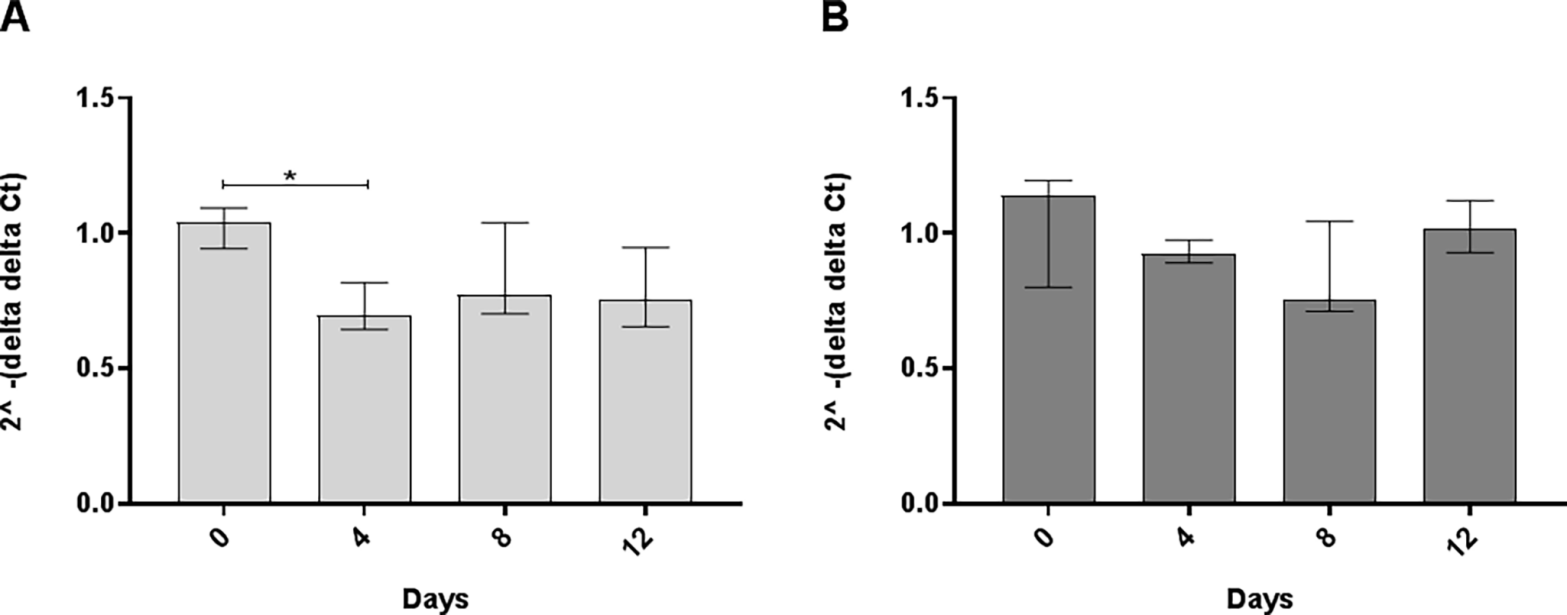
Gene expression of *Bank1*. Gene expression in spleens obtained from female A.SW and B10.S mice exposed to 8 mg HgCl_2_/L for 4, 8, or 12 days. Day 0 represents unexposed mice. Total RNA expression of *Bank1* **A**) A.SW and **B**) B10.S. Figure represents fold difference (y-axis) and presented as median ± interquartile range for each group (Kruskal Wallis, Dunn's multiple comparisons test). * Significant difference (p <0.05) between groups in each strain. *Gapdh* and *Ppia* were used as endogenous control. Fold change is relative to the mean of unexposed A.SW mice (reference sample) for the A.SW strain and the mean of unexposed B10.S mice (reference sample) for the B10.S strain.

Since Bank1 is a B-cell specific protein (no expression in T-cells) [24, 28] and *Bank1* and *Nfkb1* genes are associated with the development of higher ANoA titer, we investigated the intracellular pathway by studying gene expression in spleen and believe that our findings mainly resemble what occurs in B-cells. We were interested to see if the gene expression of these proteins (NF-kappaB and Bank1) were affected by Hg-induced ANoA and found that the *Bank1* gene expression was significantly lower in the A.SW strain (Fig 4A) on day 4 (p < 0.05), whereas its expression in the B10.S strain was not affected (Fig 4B). The lower expression of *Bank1* upon Hg-exposure may lead to dysregulation of the B-cell receptor (BCR) downstream signaling pathway, which is regulated by Bank1 [24, 33].

**Fig 5:**
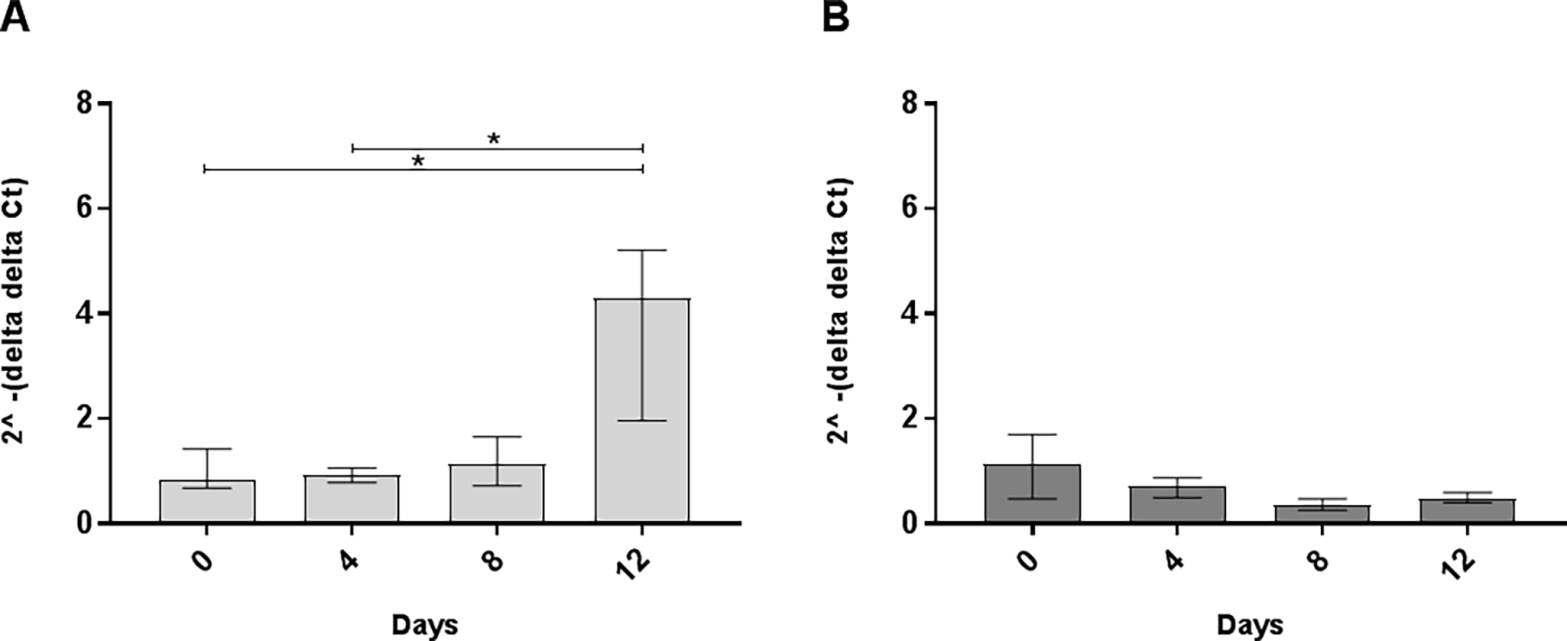
Gene expression of *Nfkb1*. Experimental design same as in Fig 4. Total RNA expression of *Nfkb1* in **A**) A.SW and **B**) B10.S mice. Figure represents fold difference (y-axis) and presented as median ± interquartile range for each group (Kruskal Wallis, Dunn's multiple comparisons test). Significant difference (* p < 0.05, ** p < 0.01) between groups in each strain. Endogenous controls and statistical analysis same as in Fig 4.

One key protein in the intracellular cascade is NF-kappaB, which we also found to be associated with ANoA development. Upon BCR signaling, downstream signaling leads to the activation of NF-kappaB-induced expression of various cytokines, such as Il-6 and Tnfα [34]. We found that *Nkfb1* gene expression was significantly higher after 12 days of Hg-exposure compared to day 0 and day 4 (p < 0.05) in the A.SW strain (Fig 5A). The less-susceptible B10.S strain showed no significant difference in the *Nfkb1* expression after Hg-exposure (Fig 5B).

**Fig 6:**
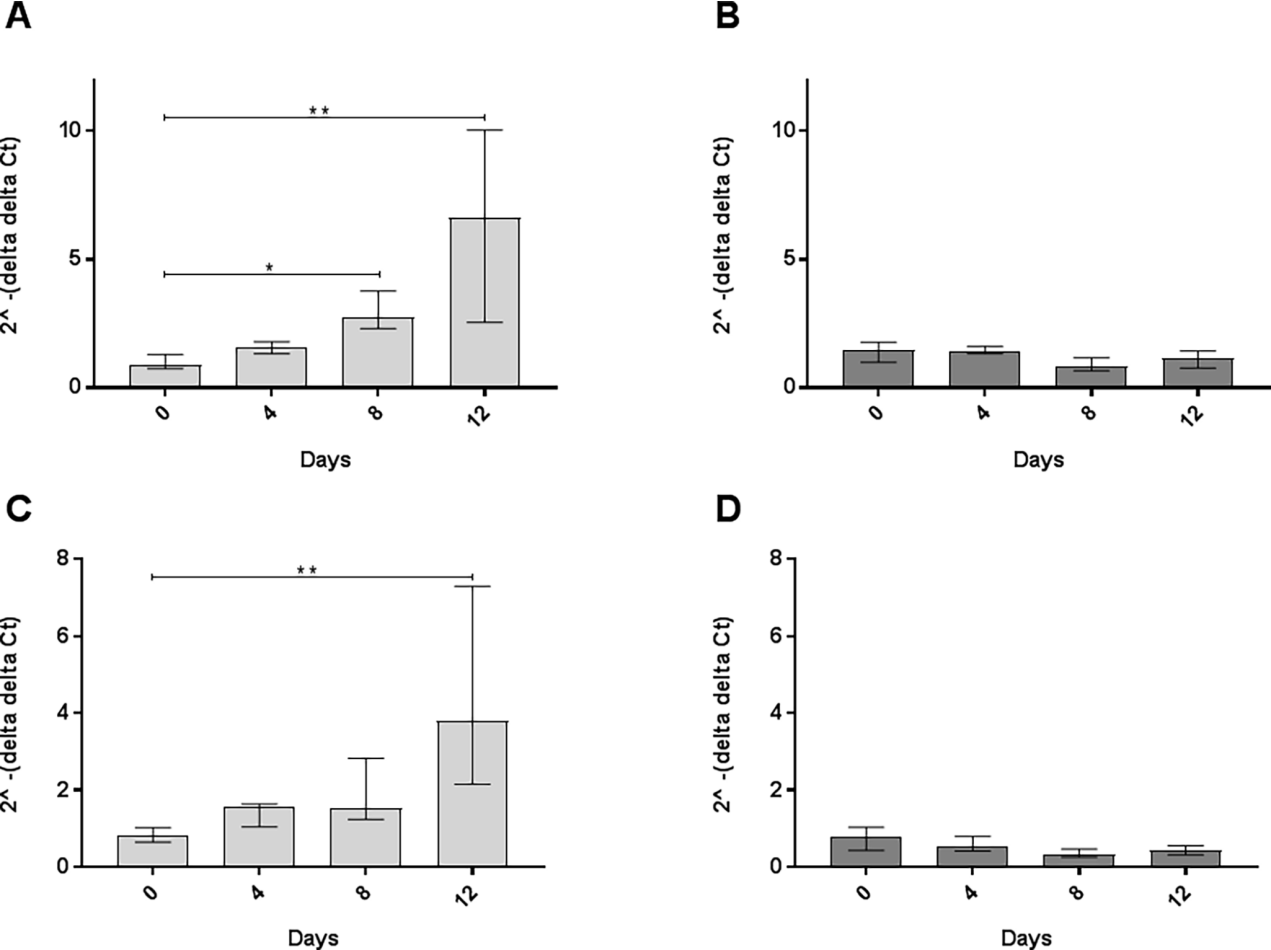
Gene expression of *Il6* and *Tnf*. Experimental design same as in Fig 4. Total RNA expression of *Il6* in **A**) A.SW and **B**) B10.S mice followed by *Tnf* expression in **C)** A.SW and **D)** B10.S mice. Figure represents fold difference (y-axis) and presented as median ± interquartile range for each group (Kruskal Wallis, Dunn's multiple comparisons test). Significant difference (* p < 0.05, ** p < 0.01) between groups in each strain. Endogenous controls and statistical analysis same as in Fig 4.

Further, the *Il6* and *Tnf* gene expression in the A.SW strain was significantly increased (p <0.05) on days 8 and 12 compared to day 0 (Fig 6A and 6C). The B10.S showed no differences in *Il6* and *Tnf* gene expression at all time-points except for *Il6* between days 4 and 8 (p = 0.0087) (Fig 6B and 6D). IL-6 regulates multiple biological processes and is highly involved in autoimmunity [20, 35–40]. In humans, IL-6 is elevated in both SLE [35] and Ssc [36]. RA patients show high IL-6 concentrations in synovial fluids [37], and treatment with anti-IL-6 receptor antibodies responds effectively for some rheumatic patients [38]. In mice, Il-6 exacerbate disease activity by activation of Th-1 cells in the EAE mouse model [39], and of Th-17 cells in Salmonella-infected mice [40]. We have previously shown that in *Il-6* KO mice HgIA abrogates the development of ANoA [20]. We believe that the susceptibility for developing ANoA is regulated by the H-2 loci, but the enhancement is regulated by an intrinsic BCR pathway regulated by Bank1 and NF-kappaB. This leads to expression of cytokines, such as IL-6 and Tnfα, that are highly involved in autoimmunity.

### Splice variants of Bank1 are associated with ANoA

**Fig 7:**
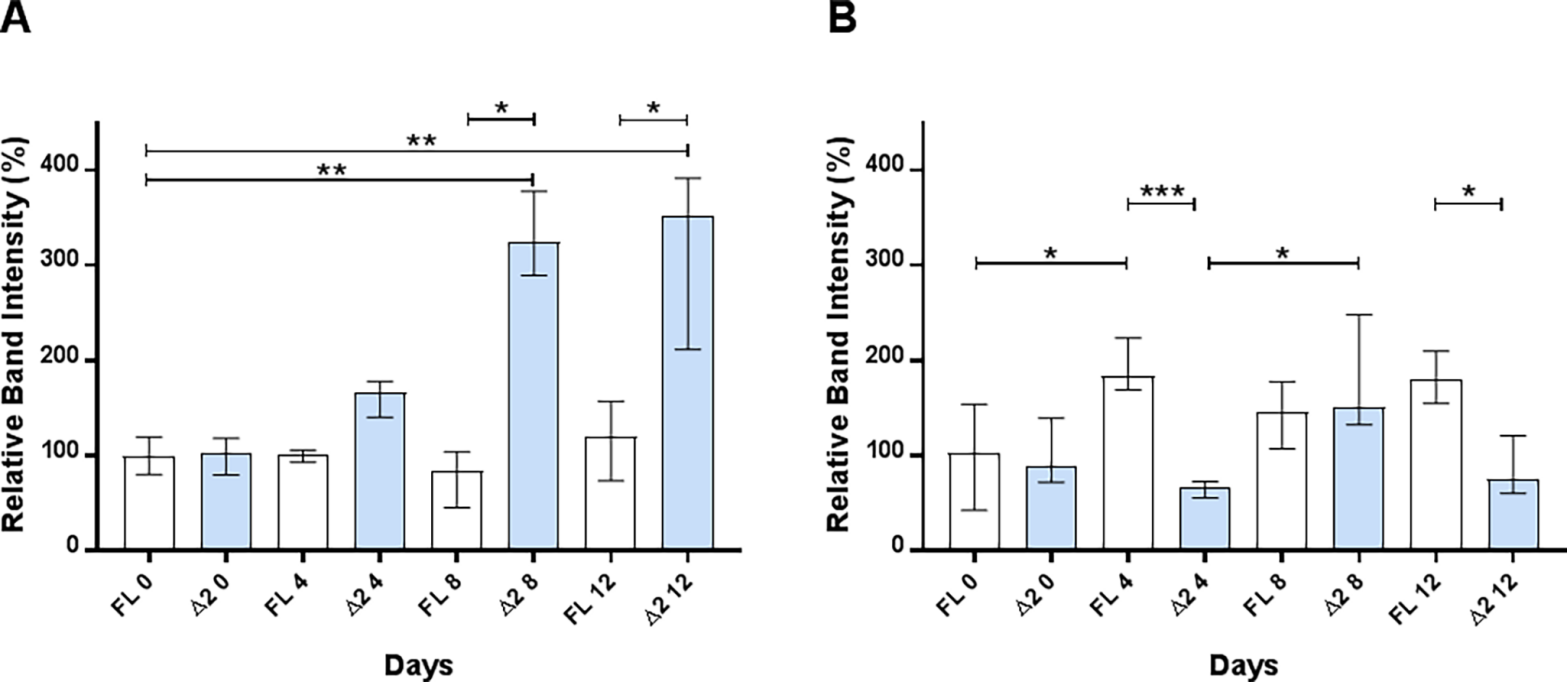
Relative total RNA expression of splice variants of Bank1. Relative total RNA expression of splice variants of *Bank1* in spleens. Experimental design is same as in Fig 4. Figure represents relative RNA expression (y-axis) of full length and Δ2 splice variants in **A)** A.SW and **B)** B10.S. Empty bars represents full-length (FL) splice variant and filled bar represents short length (Δ2) splice variant. Figure presented as median ± interquartile range for each group (Kruskal Wallis, Dunn's multiple comparisons test). Significant difference (** p < 0.01) between groups in each strain.

Bank1 consists of two splice variants, one full-length and another, Δ2, lacking exon 2. Increased quantities of the full-length isoform compared to the Δ2 isoform are associated with higher risk of systemic autoimmune disease development in humans [41]. The reduced amount of the Δ2 isoform, compared to full-length isoform, is correlated with a s10516487 R61 risk variant in humans [42]. We studied the splice variant expression in our two strains, since these strains are susceptible to ANoA production and the A.SW strain shows a higher ANoA titer compared to the B10.S strain. To detect splice variants, the relative gel band intensities of amplified DNA fragment covering exon 2 were measured for A.SW and B10.S strains after 4, 8 and 12 days of Hg-exposure, with day 0 as a control group (Fig 7). Both strains expressed the full-length *Bank1* DNA fragment (with exon 2), and the short Δ2 isoform (without exon 2) (S4 Fig), which is consistent with previous findings that mice (C57BL/6, BALB/cJ, NOD/Lt, DBA/1J, NZBWF1, NZW/LacJ and NZB/BINJ) express both variants [41].

We analyzed the time-dependent expression of same splice variant and the differences in quantities between the two splice variants. The band intensity of the full-length variant in the A.SW strain showed no significant difference on all days when exposed to Hg (Fig 7A). The B10.S strain showed a significant increase (p < 0.05) after 4 days of Hg-exposure compared to day 0 (Fig 7B). However, the relative band intensity of the short Δ2 isoform of *Bank1* was significantly higher in the A.SW strain upon Hg-exposure (day 8; p < 0.01, day 12; p < 0.01) (Fig 7C). The less-susceptible B10.S strain showed a significant increase between days 4 and 8 (p < 0.05) (Fig 7D). Our results show that the higher expression of the short Δ2 isoform of *Bank1* is associated with a higher ANoA development. When comparing the quantities between the two splice variants, we detected a significantly higher expression of the short Δ2 isoform compared to the full-length isoform after 8 and 12 days (p < 0.05) in the A.SW strain. The B10.S strain had significantly lower expression of the short Δ2 isoform compared to the full-length isoform after 4 and 12 days (p < 0.05). In humans, the full-length splice variant is associated with SLE, and this may be explained by the higher diversity in human genes and by the higher diversity between human populations compared to the two inbred mouse strains used in this study. The data for the susceptible B10.S strain are in concordance with what was found in human SLE patients, but we also found that higher expression of the short Δ2 isoform of *Bank1* is associated with a higher ANoA titer.

### HgIA alters *Tlr9* gene expression

**Fig 8:**
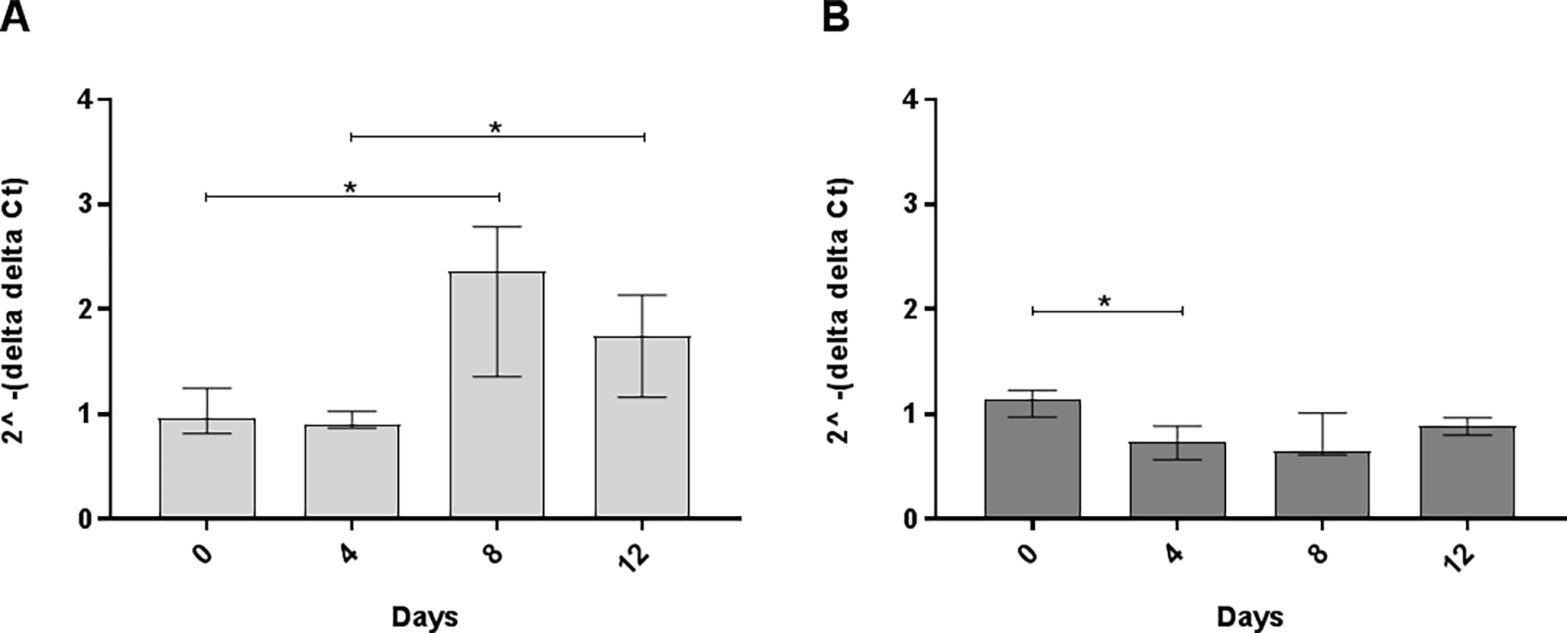
Gene expression of *Tlr9*. Experimental design same as in Fig 4. Total RNA expression of *Tlr9* in female **A**) A.SW and **B**) B10.S. Figure represents fold difference (y-axis) and presented as median ± interquartile range for each group (Kruskal Wallis, Dunn's multiple comparisons test). Significant difference (* p < 0.05) between groups in each strain. Endogenous controls and statistical analysis same as in Fig 4.

When BCR is stimulated, it also undergoes endocytosis and activates Tlr9 [43], leading to p38, JNK, and NF-kappaB activation [44]. Experiments on B-cells from *Bank1*-deficient mice showed that Bank1 controls Tlr9 signaling via the p38-MNK1/2 pathway, in which *Bank1*-deficient showed higher *Il6* expression [45]. We were therefore interested if HgIA affects the gene expression of *Tlr9*. The A.SW strain showed significantly higher *Tlr9* mRNA expression after 8 days (p = 0.043) compared to day 0 (Fig 8A). The less susceptible B10.S strain presented an opposite trend, with significantly lower *Tlr9* gene expression after 4 days (p < 0.05) compared to day 0 (Fig 8B). Taken together, Tlr9 activation and downstream signaling are involved in HgIA, where a high ANoA titer favors the Tlr9-stimulated pathway.

### A high ANoA titer seems to be initiated by BCR activation

A relevant question is, what activates the BCR to initiate the autoimmune manifestation in HgIA? Mercury has a high capacity of binding to thiol-containing proteins and affecting their structure [46]. Fibrillarin is a nucleolar protein involved in a small nucleolar ribonucleoprotein (snoRNP) complex, which is required for pre-rRNA processing [47, 48]. This protein is known to be modified by HgIA in susceptible mice and to develop ANoA that seems to target fibrillarin [49]. It has been suggested that cryptic epitopes of fibrillarin are shown as antigens by MHC class II in antigen-presenting cells (APC) presented to T-cells [49]. Our findings indicate that high ANoA development is initiated by BCR signaling since the associated genes (*Bank1* and *Nfkb1*) are linked to ANoA, and Bank1 is highly expressed in B-cells but not in T-cells [24, 28].

However, both B- and T-cells are essential for development of ANoA in HgIA [23]. We believe that autoantigens developed in HgIA may provide ligands for BCR on B-cells, which in turn function as antigen presenting cells (APC)s for T-cells by presenting their antigens via MHC class II on TCR. B-cells also activate T-cells in a variety of other ways. B-cell derived IL-6 has been shown to promote differentiation into T-follicular helper [50] cells, and activation of T helper 1 and T helper 17 cells in autoimmune disease [40, 51]. B- and T-cells are required for development of ANoA [23], but the high titer may be initiated by a BCR activation.

### What may cause a low ANoA titer?

The B10.S (H-2^*s*^) strain is also a susceptible strain to HgIA, but it initiates a less severe autoimmune manifestation with the development of lower ANoA titer compared to the A.SW strain [17, 21–23]. A relevant question is, why did the B10.S strain show a significant increase neither in the gene expression of intracellular proteins involved in the expression of Il-6 and Tnfα nor in the gene expression of these two cytokines? One explanation could be that the initiation and activation of the immune system, when exposed to Hg, may need more time. We have seen that ANoA in A.SW mice starts to develop after 12 days, whereas in the B10.S strain, the development starts after 30 days (data not shown). Another explanation could be that there are other pathways leading to the development of ANoA as well, but the BCR/Bank1/NF-kappaB pathways seem to be linked to a more severe autoimmune response.

## Materials and methods

### Mice

Mice were housed at the Animal Facilities, Linköping University, Sweden, and were kept under a controlled environment with 2–4 mice/cage. The mice were offered standard mouse pellets (CRME rodent, Special Diets services) and drinking water ad libitum. Studies were approved by the Laboratory Animal Ethics Committee, Linköping, Sweden, and all mice were treated humanely with regard to alleviating any suffering. Numbers of mice in each study are presented in Table 2.

**Table 2:** Dose of Hg exposure and number of mice in each study.

**Genome Wide Association Study**		
***Experiment***	***Number of Mice/strain***	***Mercury Exposure (mg HgCl***_***2***_***/L)***
SNP Genotyping	129 / F2	4
Haplotyping	129 / F2	4
Fine Mapping	30 / F2	4
ANoA Hg exposed	129 / F2	4
ANoA control	14 / F2	0
**Expression Study**		
***Experiment***	***Number of Mice/strain***	***Mercury Exposure (mg HgCl***_***2***_***/L)***
Gene Expression, Splice variant expression	6 per group / A.SW	8
	6 per group / A.SW	0
	6–7 per group / B10.S	8
	5 per group/ B10.S	0

#### Genetic linkage study

Female A.SW mice were obtained from Taconic, and male B10.S mice were obtained from the Jackson Laboratory. F1 hybrids were derived by crossing female A.SW mice and male B10.S mice. F2-hybrids (n = 60 males, 83 females) were obtained by crossing F1-hybrids. F2 mice (8–10 weeks old) received either tap water (n = 14) or 4.0 mg HgCl_2_/L (Fluka) in drinking water (n = 129), for 6 weeks before sacrifice. Blood was obtained from retro-orbital plexus after 6 weeks, and all animals were sacrificed by cervical dislocation, and tails, spleens and kidneys were obtained and stored at -70°C. Collected blood was allowed to clot for 2 hours before being centrifuged at 500 g for 15 minutes, after which the serum was removed and subsequently stored at -20°C.

#### Expression study

Female A.SW mice were obtained from Taconic and female B10.S mice were obtained from the Jackson Laboratory. Mice (8–9 weeks old) were given 8.0 mg HgCl_2_/L (Fluka) in drinking water for 0, 4, 8 or 12 days. Each group consisted of 5–7 female mice. Mice were sacrificed by cervical dislocation, and spleens were sampled in RNAlater (Invitrogen) and stored at -70°C for subsequent analysis.

### Genetic linkage analysis

#### Serum antinuclear antibodies assessed by Indirect immunofluorescence

Sera from 129 Hg-exposed and 14 unexposed F2 mice were diluted 1:80–1:20,480 and incubated with HEp-2 cells (Binding Site Ltd, Birmingham, UK) to detect binding of goat anti-mouse IgG antibodies (Sigma, St Louis, Missouri, USA) to cellular antigens [52]. The ANoA titer was defined as the highest serum dilution that showed specific ANoA staining. No staining at serum dilution of 1:80 was considered as a negative result (0). The fluorescence intensity of ANoA was assessed blinded in a Nikon incident-light fluorescence microscope (Nikon Instech Co. Ltd., Kanagawa, Japan) using coded samples.

#### DNA extraction

DNA isolated from tail, spleen or kidney from A.SW, B10.S, F1 and F2 mice was extracted using the Wizard SV Genomic DNA Purification System (Promega). These tissues were used to obtain the required amounts and concentrations of DNA. The quantity and purity of DNA was measured with a Qubit 3.0 fluorometer (Thermo Fisher Scientific).

#### Genome-wide genotyping and quantitative trait loci (QTL)

For the genome-wide genotyping, 129 Hg-exposed F2 samples were genotyped using the SNP&SEQ technology platform at Uppsala University. The Illumina Mouse Medium Density Linkage Panel contained 1449 single-nucleotide polymorphism (SNP) markers, of which 819 were polymorphic SNPs between the two strains A.SW and B10.S (S5 Fig).

Genotype-phenotype linkage analysis in F2 mice was performed to obtain genetic positions associated with ANoA. Quantitative trait loci (QTL) were identified based on the logarithm of odds (LOD) score profiles derived from a genome-wide single-QTL scan by Haley-Knott regression [53] with a hidden Markov model (HMM) using R/qtl software (v.2.15.3) [54]. Regression was based on the data from 129 F2 offspring for 819 SNPs covering 19 autosomes. The origin of DNA determines the recommended LOD score for linkage. Outbred mice, or humans, require a higher linkage percentage compared to the inbred mice that were used in this study. The genome-wide significance threshold was calculated based on 10,000 permutation replicates. This procedure is based on the normal distribution and gives an approximate p-value of 0.05 [54, 55].

#### Haplotyping

Additional microsatellites were used to narrow down the region by haplotype analysis in which the QTL was found (S1 Table) in 129 Hg-exposed F2 mice and two unexposed A.SW and B10.S mice. Briefly, microsatellite primers were identified using the Mouse Genome Informatics (MGI) database [56] based on the background strains of A (for A.SW) and C57BL6 (for B10.S). For each sample, 20 ng of genomic DNA was mixed with the Extract-N-Amp PCR reaction mix (Sigma-Aldrich), and 30 cycles of amplification were performed in a thermal cycler (Thermo Fisher), with a temperature profile as follows: denaturation at 94°C for 30 s, annealing at 61°C for 60 s, and extension at 72 C for 90 s. PCR products were run on 4% agarose gel for 1.5 hours at 70 Volts. Haplotypes were identified by comparing the genotypes of F2 mice with the genotypes of A.SW and B10.S mice [57].

#### Fine mapping

Thirty F2 mice, homozygous for the A.SW strain on associated haplotype, were selected for fine mapping. Fine mapping was based on next generation sequencing (NGS) of genes within the haplotype containing SNPs between background strains A (for A.SW) and C57BL/6 (for B10.S). SNPs were identified using the Ensembl [29] and MGI databases [56]. Design of target sequences was performed using the web-based application SureDesign (Agilent) for coding exons and UTRs (5´UTR and 3´UTR) for 11 genes (S2 Table). The genomic DNA (gDNA) library was prepared from 30 F2 mice (homozygous for A.SW strain on marker rs3676039), one A.SW mouse and one B10.S mouse (used as controls) using SureSelect QXT Target Enrichment for Illumina kit (Agilent) in accordance with the manufacturer´s protocols. Briefly, 32 DNA samples (n = 30 for F2 mice, n = 1 for A.SW mice, n = 1 for B10.S mice) were enzymatically fragmented, and adaptors were added to the ends of the fragments (350 bp fragment size). gDNA libraries were amplified and purified, followed by hybridization and capture the next day. Libraries were indexed and pooled into 4 groups (8 libraries per group) for multiplex sequencing. Sequencing was performed with a MiSeq Benchtop Sequencer (Illumina) using 500 cycles paired-end reads and a MiSeq v2 reagent kit (Illumina). All data were analyzed using the command line in the Linux operating system. Quality score of raw data (FASTQ files) were analyzed with FastQC [58]. Sequence data were aligned with the mouse reference gene, *Mus musculus* USCS Mm10 [59], using the Burrows-Wheeler Aligner (BWA) software package [60]. Aligned sequencing data (SAM files) were converted into BAM files with SAM tools [61]. Variant calling was performed with the Genome Analysis Toolkit (GATK) [62]. Genotype data on all 30 F2 mice were used for additional linkage analysis with R/QTL.

### Expression study

#### RNA extraction and cDNA reverse transcription

Total RNA was extracted from spleens of female A.SW and B10.S mice (n = 24) using the RNeasy Mini Kit (Qiagen) according to the manufacturer’s instructions. The quantity and purity of the RNA were measured using a Qubit 3.0 fluorometer (Thermo Fisher Scientific). RNA was diluted to 20 ng/μL and reverse-transcribed to cDNA by using the High-Capacity cDNA Archive kit (Applied Biosystems).

#### Gene expression

Gene expression from A.SW and B10.S mice was performed in duplicates using the Applied Biosystems 7500 Fast Real-Time PCR system with Applied Biosystems TaqMan gene expression assays (Applied BioSystems). Target gene expression for *Bank1*, *Nfkb1*, *Tlr9*, *Il6* and *Tnf* was measured with FAM (6-carboxyfluorescein) reporter dye–labeled probes (S3 Table). The geometric means of *Gapdh* and *Ppia* in each group were used as endogenous controls. The results are presented as relative transcription levels determined by the comparative 2^–ΔΔCt^ method [57].

#### Splice variant expression

cDNA of *Bank1* from A.SW and B10.S mice, encompassing exon 2 and the upstream exon 1 and downstream exon 3 sequences, was amplified for splice variant detection in A.SW and B10.S mice (F primer: ATGCTTCCTGTGGCTTCTGG, R primer: CGAGGCACAGATGGTCTCAG). Fragments were amplified by 30 cycles of PCR under following conditions: denaturation at 94°C for 30 s, annealing at 60°C for 60 s, and extension at 72°C for 90 s. PCR products were separated on 1% agarose gel for 30 minutes at 120 Volts and measured with the GeneFlash Gel Documentation System (GeneFlash). Bands where quantified based on their relative intensities using ImageJ software 1.x [63].

### Secondary structure prediction

Prediction of the secondary structure of Bank1 protein was performed using the Chou & Fasman Secondary Structure Prediction (CFFSP) server. The cDNA sequences of background strains A.SW and B10.S were used to obtain the protein sequences that were used to predict the secondary structures of Bank1 by the Chou & Fasman algorithm [30]. The cDNA sequences were obtained from the Ensembl database [29].

### Conserved region

Comparison of SNPs in the *Bank1* gene between background strains of 32 mammals (S2 Fig) was performed using the Ensembl database (Flicek et al. 2017). The conserved region of the amino acid sequences was analyzed using Clustal X (version 2.1) multiple sequence alignment software (Larkin et al. 2007). Amino acid sequence alignment was performed for 14 species (S3 Fig) together with A (background strain for A.SW) and C57BL/6 (background strain for B10.S) mouse strains. These 14 species were selected because they have a sequenced *Bank1* gene that can be used for alignment using the Ensembl database.

### Statistical analysis

Comparisons of ANoA titers, gene expression levels and splice variants were performed using the Kruskal-Wallis and Dunn's multiple comparisons tests and presented as medians ± interquartile ranges. The effect plot was obtained using the non-parametric Mann-Whitney U-test and presented as mean ± SEM. Differences with p < 0.05 were considered significant.

## Conclusion

*Bank1* and *Nfkb1* are based on genome-wide scan and fine mapping of the candidate genes for regulation of high antinucleolar antibody titer. Mutations, gene expression and splice variant expression of *Bank1*, as well as gene expression of *Nfkb1*, are associated with the susceptibility to the development of ANoA. A high ANoA titer seems to be B-cell initiated with Bank1 and NF-kappaB as key regulators in the intracellular pathway, including Tlr9, leading to the production of cytokines such as Il-6 and Tnfα, which are highly involved in autoimmune manifestations.

## Supporting information

S1 Table Microsatellite markers that differ between background strains A and C57BL/6, used in the haplotype study in order to narrow down the QTL region. Primer sequences and amplicon sizes of A and C57BL/6 are also presented.
(DOCX)

S2 Table Design of target sequences was performed using web-based application SureDesign (Agilent). Sequencing was design for 11 genes targeting coding exons, and UTRs (5´UTR and 3´UTR).
(DOCX)

S3 Table Gene targets used in the gene expression analysis, measured with FAM labeled probes. Gene/protein names followed by gene symbols are presented.
(DOCX)

S1 Fig Secondary structure prediction for missense variants on Bank1 between the two strains A.SW and B10.S. A) Missense variant rs30260564, F75L on exon 2 and B) missense variants rs50828248 and rs47442962, A375M on exon 7. Prediction of secondary structure was performed with the Chou & Fasman algorithm by the use of the online software server Chou & Fasman Secondary Structure Prediction (CFFSP) (Ashok Kumar T 2013). Query: amino acid position, Helix: α-helix structure, Sheet: β-sheet structure, Turns: structure folding.
(DOCX)

S2 Fig Thirty-two mammalian species were selected for conserved region by using Ensembl database, which run a nucleotide alignment against rs30260564, rs50828248 and rs47442962 on *Bank1*. (DOCX)

S3 Fig Conserved amino acid sequences for rs30260564, rs50828248 and rs47442962. Fourteen mammalian species were selected by using Ensembl database for retrieval of amino acid sequences. Conserved region on amino acids were performed by aligning the multiple sequences with the use of Clustal X (version 2.1). rs50828248 and rs47442962 code for the same amino acid.
(DOCX)

S4 Fig cDNA fragments of Bank1 splice variants obtained from A.SW and B10.S mice exposed to 8 mg HgCl2/L for 4, 8, or 12 days. Day 0 represents unexposed mice. Amplified cDNA encompass exon 2 and the upstream exon 1 and downstream exon 3 sequence. Full-Length represents cDNA with exon 2 and Delta 2 represents cDNA lacking exon 2.
(DOCX)

S5 Fig Genetic map of F2 population showing physical location of informative autosomal SNP markers. For an SNP to be informative, it should vary between the genotypes of the A.SW and B10.S strains.
(DOCX)
