# Supplementary material for: Bank1 and NF-kappaB as key regulators in anti-nucleolar antibody development
Source: PLoS One. 2018 Jul 17;13(7):e0199979. doi: 10.1371/journal.pone.0199979 (PMC6049909; doi:10.1371/journal.pone.0199979)
Supplement: S1 Table — Primer sequences and amplicon sizes of A and C57BL/6 are also presented. (DOCX) [file pone.0199979.s001.docx]

**S1 Table. Microsatellite markers used for haplotyping**

| Markers | Chr | cM | Direction | Primer Pairs | A | C57BL/6 |
| --- | --- | --- | --- | --- | --- | --- |
| D3Mit216 | 3 | 54,48 | F | AGGACTGAAGAAACATACACATGC | 142 | 122 |
|  |  |  | R | AGAAACATCTTGATTTTCAACAAGG |  |  |
| D3Mit318 | 3 | 54,58 | F | CTCATTCCTTCTGAGCAATGG | 162 | 146 |
|  |  |  | R | TATGGGATATGCTTTTCATAAAAGG |  |  |
| D3Mit159 | 3 | 55,85 | F | CTAACCCAGATAGGGCTTTGG | 154 | 152 |
|  |  |  | R | AGATTACCCTCTGGTGTTTACATACA |  |  |
| D3Mit348 | 3 | 56,07 | F | CATCATGCATACTTTTTTCCTCA | 108 | 124 |
|  |  |  | R | GCCAAATCATTCACAGCAGA |  |  |
| D3Mit109 | 3 | 56,54 | F | TGTACTGCTCCCAAGCACAC | 246 | 234 |
|  |  |  | R | ACCTTCATTTATTGGATGTGTTATACA |  |  |
| D3Mit247 | 3 | 56,63 | F | ACCTTCCCACATACATCTCCA | 178 | 136 |
|  |  |  | R | TACTGACTGAGCAGATTATATTTGGG |  |  |
| D3Mit319 | 3 | 56,89 | F | TCTCCCTCACTTTTTCCTTCC | 193 | 177 |
|  |  |  | R | AACAGCCAGTCCAGCAAATC |  |  |
| D3Mit145 | 3 | 57,70 | F | TCTCGATGTGCACATGTCTG | 147 | 149 |
|  |  |  | R | TTTCTATCATTTAGTGCTTAGGAGAGG |  |  |
| D3Mit254 | 3 | 61,32 | F | TTCAGCATGTGTCCACCATT | 138 | 150 |
|  |  |  | R | CACATTATTTTGAATTTCTAGGTAGGG |  |  |

Microsatellite markers that differ between background strains A and C57BL/6, used in the haplotype study in order to narrow down the QTL region. Primer sequences and amplicon sizes of A and C57BL/6 are also presented.
