# Supplementary material for: Bank1 and NF-kappaB as key regulators in anti-nucleolar antibody development
Source: PLoS One. 2018 Jul 17;13(7):e0199979. doi: 10.1371/journal.pone.0199979 (PMC6049909; doi:10.1371/journal.pone.0199979)
Supplement: S2 Table — Sequencing was design for 11 genes targeting coding exons, and UTRs (5´UTR and 3´UTR). (DOCX) [file pone.0199979.s002.docx]

**S2 Table.** **Design detail of target sequencing for Next Generation Sequencing (NGS)**

| **Gene** | **Target ID** | **Regions** | **Covarage** |
| --- | --- | --- | --- |
| Lef1 | ENSMUSG00000027985 | 13 | 98.373344 % |
| Ccdc109b | ENSMUSG00000027994 | 8 | 100.0 % |
| Ppa2 | ENSMUSG00000028013 | 14 | 100.0 % |
| Egf | ENSMUSG00000028017 | 25 | 100.0 % |
| Dkk2 | ENSMUSG00000028031 | 4 | 100.0 % |
| Nfkb1 | ENSMUSG00000028163 | 26 | 98.30636 % |
| Manba | ENSMUSG00000028164 | 21 | 97.970505 % |
| Bank1 | ENSMUSG00000037922 | 17 | 100.0 % |
| Elovl6 | ENSMUSG00000041220 | 5 | 100.0 % |
| Slc9b1 | ENSMUSG00000050150 | 14 | 95.11212 % |
| Col25a1 | ENSMUSG00000058897 | 40 | 100.0 % |

Design of target sequences was performed using web-based application SureDesign (Agilent). Sequencing was design for 11 genes targeting coding exons, and UTRs (5´UTR and 3´UTR).
