# Supplementary material for: Bank1 and NF-kappaB as key regulators in anti-nucleolar antibody development
Source: PLoS One. 2018 Jul 17;13(7):e0199979. doi: 10.1371/journal.pone.0199979 (PMC6049909; doi:10.1371/journal.pone.0199979)
Supplement: S3 Table — Gene/protein names followed by gene symbols are presented. (DOCX) [file pone.0199979.s003.docx]

**S3 Table. Gene targets for Real-Time PCR**

| **Gene/Protein name** | **Gene Symbols** | **Species** | **Dye Label** | **Assay ID** |
| --- | --- | --- | --- | --- |
| B-cell scaffold protein with ankyrin repeats 1 | *Bank1* | Mouse | FAM | Mm01317739_m1 |
| Toll like receptor 9 | *Tlr9* | Mouse | FAM | Mm00446193_m1 |
| Interleukin 6 | *Il6* | Mouse | FAM | Mm00446190_m1 |
| Tumor necrosis factor | *Tnf* | Mouse | FAM | Mm00443258_m1 |
| Peptidylprolyl isomerase A | *Ppia* | Mouse | FAM | Mm02342430_g1 |
| Glyceraldehyde-3-phosphate dehydrogenase | *Gapdh* | Mouse | FAM | Mm99999915_g1 |

Gene targets used in the gene expression analysis, measured with FAM labeled probes. Gene/protein names followed by gene symbols are presented.
