# Supplementary material for: Bank1 and NF-kappaB as key regulators in anti-nucleolar antibody development
Source: PLoS One. 2018 Jul 17;13(7):e0199979. doi: 10.1371/journal.pone.0199979 (PMC6049909; doi:10.1371/journal.pone.0199979)
Supplement: S1 Fig — A) Missense variant rs30260564, F75L on exon 2 and B) missense variants rs50828248 and rs47442962, A375M on exon 7. Prediction of secondary structure was performed with the Chou & Fasman algorithm by the use of the online software server Chou & Fasman Secondary Structure Prediction (CFFSP) (Ashok Kumar T 2013). Query: amino acid position, Helix: α-helix structure, Sheet: β-sheet structure, Turns: structure folding. (DOCX) [file pone.0199979.s004.docx]

**S1 Fig.** **Prediction of Secondary Structure of Bank1.**

**Exon 2**

B10.S


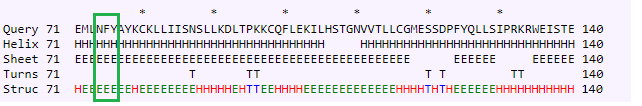


A.SW


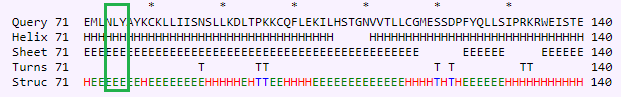


**Exon 7**

B10.S


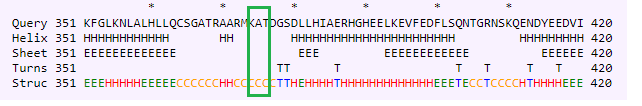


A.SW


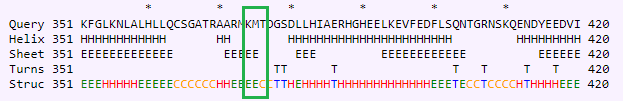


Secondary structure prediction for missense variants on Bank1 between the two strains A.SW and B10.S. A) Missense variant rs30260564, F75L on exon 2 and B) missense variants rs50828248 and rs47442962, A375M on exon 7. Prediction of secondary structure was performed with the Chou & Fasman algorithm by the use of the online software server Chou & Fasman Secondary Structure Prediction (CFFSP) (Ashok Kumar T 2013). Query: amino acid position, Helix: α-helix structure, Sheet: β-sheet structure, Turns: structure folding.
