# Supplementary material for: Bank1 and NF-kappaB as key regulators in anti-nucleolar antibody development
Source: PLoS One. 2018 Jul 17;13(7):e0199979. doi: 10.1371/journal.pone.0199979 (PMC6049909; doi:10.1371/journal.pone.0199979)
Supplement: S2 Fig — (DOCX) [file pone.0199979.s005.docx]

**S2 Fig.** **Conserved nucleotide region of SNPs on Bank1.**

rs30260564 – A (**T**), C57BL/6 (**G**)

Mouse C57BL/6 strain TGTAAGCATA **G** AAGTTTAGCA

Mouse A strain TGTAAGCATA **T** AAGTTTAGCA

Rat TGTAAGCATT **T** AAGTGTAGCG

Panda TGTAAGAGTT **T** AAACTCAGCA

Ferret TGTAAGAGTT **T** AAATTCAGCA

Dog TATAACAGTT **T** AAACTCAGCA

Cat TGTAAGAGTT **T** AAACTCAGCA

Cow TGTAGGAGCT **T** AAATTCAGCA

Sheep TGTAGGAGCT **T** AAATTCAGCA

Pig TGTAGGAGTT **T** AAATCCAGCA

Dolphin TGTAAGAGTT **T** AAACTTAGCA

Horse TGTAAGAGTT **T** AAACTCAGCA

Microbat TGTAAGAGTT **T** AAACTCAGCA

Megabat TGTAGGAATT **T** AAACTCAGCA

Armadillo TGTAAGAGTT **T** AAACTCAGTA

Marmoset TGTAAAAGGT **T** AAGTTCAGCA

Vervet-AGM TGTAAGAGGT **T** AAGTTCAGCA

Macaque TGTAAGAGGT **T** AAGTTCAGCA

Olive baboon TGTAAGAGGT **T** AAGTTCAGCA

Gorilla TGTAAGATGT **T** AAGTTCAGCA

Human TGTAAGACGT **T** AAGTTCAGCA

Chimpanzee TGTAAGACGT **T** AAGTTCAGCA

Orangutan TGTAAGAGGT **T** AAGTTCAGCA

Gibbon TGTAAGAGGT **T** AAGTTTAGCA

Bushbaby TGTAGGAGGT **T** AAGTCCAGCA

Tarsier TGTAAGAGGT **T** AAGTTCAGCA

Tree Shrew TGTAAGAGTG **T** AAGCTTAGCA

Rabbit TGTAAGAATT **T** AAGCTCATTA

Squirrel TGTAAGAGTT **T** AAGCTTAGCA

Guinea Pig TGTAAGAGGG **T** AAGCCTAACA

Kangaroo rat TGTAAGAATT **T** AAACTTAGCA

Elephant TGTAAGAGTC **C** AAACTCAGCA

Hyrax TGTAAGAGCT **C** AAGCTCAGTA

rs47442962 – A (**A**), C57BL/6 (**G**) / rs50828248 – A (**T**), C57BL/6 (**C**)

62 48

Mouse C57BL/6 strain CCATCTGTC **G C** CTTCATTCTA
Mouse A strain CCATCTGTC **A T** CTTCATTCTA

Rat CCATCTGTA **G A** CTTCGTCCTA

Kangaroo rat CCATCTGTG **T T** TTTGGTCTGA

Squirrel CCATCCATA **T T** TTTCATCTTA

Guinea Pig CCACTTGTA **T T** TTTCACCTTC

Rabbit CCTTCCTTA **T T** TTTCACCTGA

Marmoset CCCTCCATA **T T** TTTCATCTGA

Vervet-AGM CCCTCCATA **T T** TTTCATCTTA

Macaque CCCTCCATA **T T** TTTCATCTTA

Olive baboon CCCTCCATA **T T** TTTCATCTTA

Human CCCTCCATA **T T** TTTCATCTTA

Chimpanzee CCCTCCGTA **T T** TTTCATCTTA

Orangutan CCCTCCATA **T T** TTTCATCTTA

Gibbon CCCTCCATA **T T** TTTCATCTTT

Tarsier ACTTCTGTA **T T** TTTCATCTTA

Bushbaby CCTTCTGTA **T T** TTTCACCTTA

Tree Shrew CCCTCCATA **T T** TTTCATCTGC

Panda CCCTCGAAA **T T** TTTCATCTTA

Ferret CCCACGAAA **T T** TTTCATCTTA

Dog CCCTCGAAA **T T** TTTCATCTCA

Cat CCCTCCAAA **T T** TTTCACCTTA

Horse CTCTCCAAA **T T** TTTCATCTTA

Microbat CCCTCCACA **T T** TTTTATCTGA

Megabat CCCTCCAAA **T T** TTTCATTTTA

Cow CCTTCCAAA **T T** TTTTATCTTA

Sheep CCTTCCAAA **T T** TTTTATCTTA

Dolphin CCCTCCAAG **T T** TTTCACCTTA

Pig CCCTCCAAG **T T** TTTTATCTTA

Hedgehog CCGTGCTTG **T T** GGCCACACTG

Armadillo CCCTCCATA **T T** TTTCATCTGA

Elephant CCCTCCATA **T T** TTTCATCTTA

Hyrax CCATCCACG **T T** TTTCATCTGG

Thirty-two mammalian species were selected for conserved region by using Ensembl database, which run a nucleotide alignment against rs30260564, rs50828248 and rs47442962 on *Bank1*.
