# Supplementary material for: Bank1 and NF-kappaB as key regulators in anti-nucleolar antibody development
Source: PLoS One. 2018 Jul 17;13(7):e0199979. doi: 10.1371/journal.pone.0199979 (PMC6049909; doi:10.1371/journal.pone.0199979)
Supplement: S3 Fig — Fourteen mammalian species were selected by using Ensembl database for retrieval of amino acid sequences. Conserved region on amino acids were performed by aligning the multiple sequences with the use of Clustal X (version 2.1). rs50828248 and rs47442962 code for the same amino acid. (DOCX) [file pone.0199979.s006.docx]

**S3 Fig.** **Conserved amino acid region of SNPs on Bank1.**

rs30260564


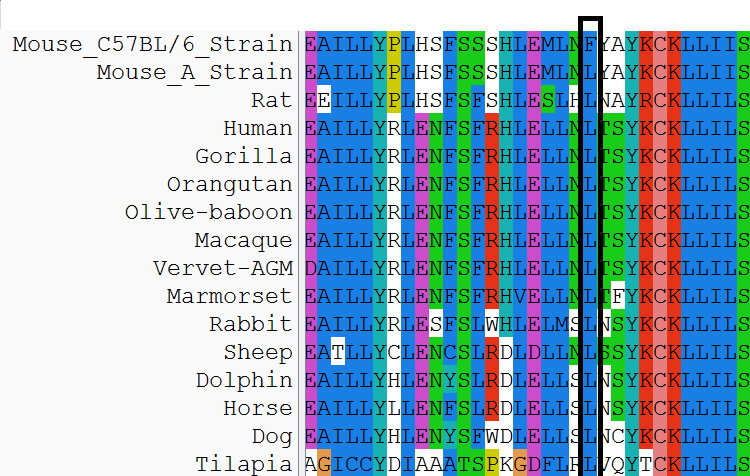


rs50828248 and rs47442962


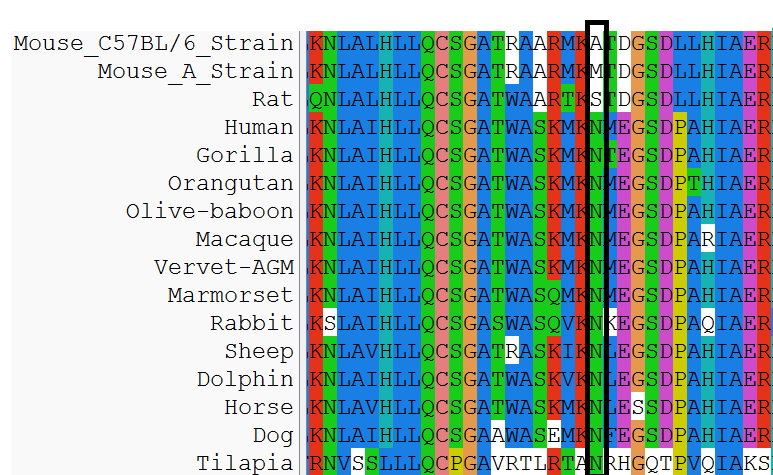


Conserved amino acid sequences for rs30260564, rs50828248 and rs47442962. Fourteen mammalian species were selected by using Ensembl database for retrieval of amino acid sequences. Conserved region on amino acids were performed by aligning the multiple sequences with the use of Clustal X (version 2.1). rs50828248 and rs47442962 code for the same amino acid.
