# Supplementary material for: Bank1 and NF-kappaB as key regulators in anti-nucleolar antibody development
Source: PLoS One. 2018 Jul 17;13(7):e0199979. doi: 10.1371/journal.pone.0199979 (PMC6049909; doi:10.1371/journal.pone.0199979)
Supplement: S4 Fig — Day 0 represents unexposed mice. Amplified cDNA encompass exon 2 and the upstream exon 1 and downstream exon 3 sequence. Full-Length represents cDNA with exon 2 and Delta 2 represents cDNA lacking exon 2. (DOCX) [file pone.0199979.s007.docx]

**S4 Fig. cDNA fragments of Bank1 splice variants.**

A.SW mice


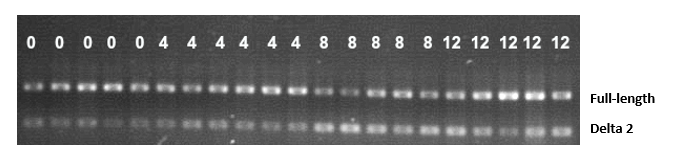


B10.S mice


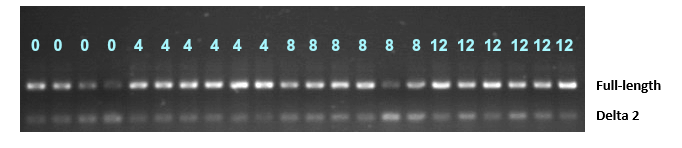
 cDNA fragments of Bank1 splice variants obtained from A.SW and B10.S mice exposed to 8 mg HgCl2/L for 4, 8, or 12 days. Day 0 represents unexposed mice. Amplified cDNA encompass exon 2 and the upstream exon 1 and downstream exon 3 sequence. Full-Length represents cDNA with exon 2 and Delta 2 represents cDNA lacking exon 2.
