# Supplementary material for: Bank1 and NF-kappaB as key regulators in anti-nucleolar antibody development
Source: PLoS One. 2018 Jul 17;13(7):e0199979. doi: 10.1371/journal.pone.0199979 (PMC6049909; doi:10.1371/journal.pone.0199979)
Supplement: S5 Fig — For an SNP to be informative, it should vary between the genotypes of the A.SW and B10.S strains. (DOCX) [file pone.0199979.s008.docx]

**S5 Fig. Genetic Map**


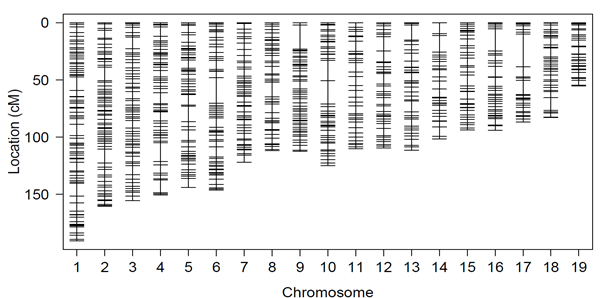


Genetic map of F2 population showing physical location of informative autosomal SNP markers. For an SNP to be informative, it should vary between the genotypes of the A.SW and B10.S strains.
